# Stafne’s bone cavity: An unusual case with involvement of the buccal and lingual mandibular plates

**DOI:** 10.4317/jced.51229

**Published:** 2014-02-01

**Authors:** Judit Herranz-Aparicio, Rui Figueiredo, Cosme Gay-Escoda

**Affiliations:** 1 DDS. Fellow of the Master of Oral Surgery and Implantology. School of Dentistry. University of Barcelona, Spain; 2 DDS. Associate Professor of Oral Surgery. Professor of the Master of Oral Surgery and Implantology. School of Dentistry. University of Barcelona. IDIBELL Research group, Barcelona, Spain; 3 MD, DDS, PhD. Chairman and Professor of Oral and Maxillofacial Surgery. Director of the Master of Oral Surgery and Implantology. School of Dentistry.University of Barcelona. IDIBELL Research group. Oral and Maxillofacial surgeon at the Teknon Medical Center, Barcelona, Spain

## Abstract

Lingual mandibular bone defects, also known as Stafne bone cavity (SC), are unilateral asymptomatic radiolucencies, generally seen in the mandibular angle, below the inferior alveolar canal. Although panoramic radiographies normally offer enough information to make a correct diagnosis, additional studies are often required, especially in atypical cases. The present report describes an atypical presentation of a Stafne’s bone cavity in a 78 years-old male patient. In this particular case, an asymptomatic and radiolucid lesion was observed during a routine dental examination. The computed tomography (CT) showed an involvement of both lingual and buccal mandibular plates producing a tunnel-like lesion. No history of mandibular trauma or surgery was refered. An additional magnetic resonance imaging (MRI) was made to discard submandibular gland pathology and to confirm the diagnosis. Since SC is asymptomatic and nonprogressive, a conservative approach based in clinical and radiological follow-ups was considered to be the most suitable treatment option.

** Key words:**Stafne bone cavity, lingual mandibular bone defect, case report.

## Introduction

The Stafne bone cavity (SC) was first described in 1942 by Edward Stafne, which reported 35 cases of unilateral, radiolucent and asymptomatic cavities located between the lower first molar and the mandibular angle (1). Since then, many terms have been used to describe this entity: Stafne bone cyst, Stafne bone cavity, static bone cavity, latent bone cyst, developmental bone defect of the mandible, lingual mandibular salivary gland depression, aberrant salivary gland defect, lingual cortical mandibular bone defect, submaxillary salivary gland inclusion, among others (2,3).

The patients with SC are usually asymptomatic and therefore the diagnosis is frequently made on routine oral radiographic examinations (3-6). Although some reports describe cases with bilateral presentation or with ante-rior defects, typically these lesions are unilateral and are located in the posterior area of the mandible, below the mandibular canal (2,5). The differential diagnosis of these cases is usually straightforward although some difficulties may appear when this lesion is located near the apical areas of the mandibular teeth (5).

SC are mostly seen in middle aged men, usually in their fifth to seventh decades of life, and the prevalence ranges from 0,10% to 0,48%. Despite the many existing theories, up to now, its etiopathogeny is not yet fully understood although most authors concur that this cavity might be related with the pressure exerted by the submandibular gland on the lingual mandibular plate (2,3,6-8). In fact, one of the main radiological features of SC is the lingual origin of the lesion, and to our knowledge no cases with buccal mandibular plate involvement have been published to date.

The present report describes an atypical presentation of a SC with a tunnel –like lesion.

## Case Report

A 78 years-old male patient was referred to the dental Hospital of the Faculty of Dentistry of the University of Barcelona to evaluate an asymptomatic, radiolucid lesion in the mandible observed in a panoramic radiography made in a routine dental examination.

The patient had a previous history of arterial hypertension, chronic bronchitis, benign prostatic hypertrophy, hypercholesterolemia and arthritis, as well as, a previous history of acetylsalicylic acid hypersensitivity. The intraoral examination revealed a poor oral condition with chronic periodontitis, multiple radicular rests, and a fistula associated to the right second upper premolar. Bilateral mandibular lingual torus were also found in the clinical examination. The patient didn’t refer any history of mandibular trauma or surgery.

**Figure 1:**
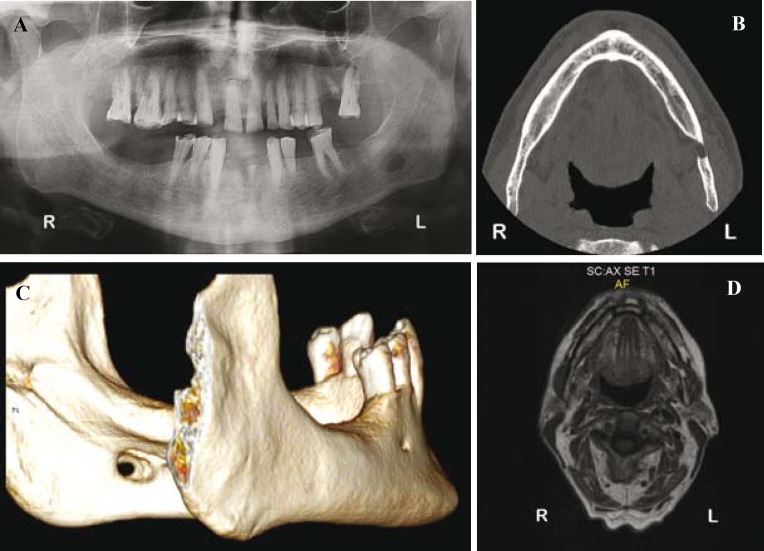
A) Panoramic radiography showing a well-defined, oval, radiolucent lesion under the left mandibular canal. B) Axial section of the CT scan of the mandible showing involvement of both lingual and buccal plates. C) 3-D reconstruction of the CT showing tunnel-type lesion. D) Coronal T1-weighted MR image. The soft tissue included in the defect is contiguous to the adjacent submandibular gland.

 In the panoramic radiography, a unilocular, ovoid, radiolucid, well-defined lesion, measuring approximately 0.6 cm x 0.9 cm, was located near the left mandibular angle below the mandibular canal (Fig. 1). No previous radiological exams were available.

In order to reveal the exact location of the defect, a computed tomography (CT) of the mandible was performed. Axial sections showed a defect, with discontinuity of the lingual plate, as well as, a significant erosion of the buccal cortex (Fig. 1). The 3D-reconstruction showed a tunnel-like lesion with a complete loss of continuity of both lingual and buccal plates (Fig. 1). Due to this last finding, a magnetic resonance imaging (MRI) was performed in order to discard submandibular gland pathology (Fig. 1).

**Figure 2:**
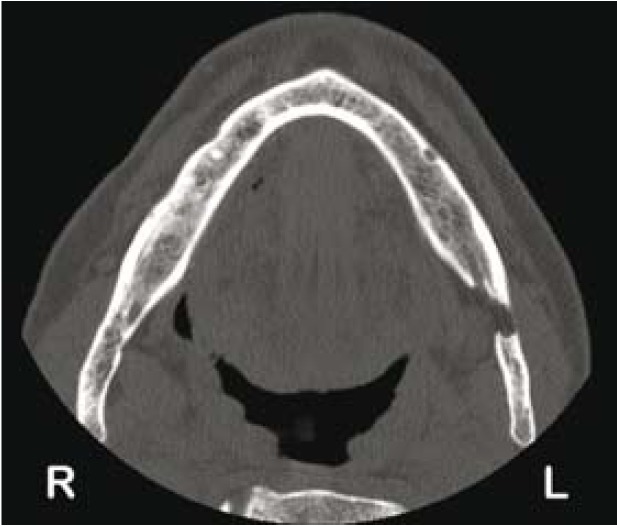
Axial section of CT scan at one year follow-up. No changes are observed with respect to the previous CT.

Although both mandibular plates were affected, a diagnosis of SC was made and no further therapy was institu-ted. A new CT was made one year after the initial diagnosis and no changes regarding the size and features of the lesion were observed (Fig. 2).

## Discussion

The typical presentation of a SC is an elliptical, homogeneous radiotransparency, with a well-defined border located below the inferior alveolar canal, often involving the lower border of the mandible (9). This lesion often contains salivary gland tissue, although some papers have showed the presence of muscular, vascular, connective and lymphoid tissues (7,9). These last findings are based in surgical reports, and may be explained by the intermittent gland herniation, regression of the herniated gland or surgical disruption of the cavity content (3,4).

The pathogenesis of the SC is not yet fully understood. The first theory, originally supported by Stafne (1), suggests that a part of the salivary gland becomes entrapped during the development and ossification of the mandible. The major objection to this etiology is that these defects are much more frequently diagnosed in adults rather than in children, suggesting that the development of such lesions probably occurs later in life, after the ossification of the mandible (5-7). Another factor that contradicts this hypothesis is that some authors have presented well-documented SC cases in which this cavity wasn’t present in previous panoramic radiographies. Mandibular bone depressions have been followed for long periods of time without evidence of size and shape changes. Accordingly to Philipsen et al. (10) a progressive reduction in the mineralised bone volume might occur making these depressions radiographically discernable on conventional radiographs around the age of 35 years.

This evolution pattern might be explained by the pressure exerted by the neighboring structures like the salivary glands (2,5,7). It is well-known that with increasing age, the major salivary glands, and in particular the submandibular gland, are the sites of nonspecific (lymphocytic) inflammatory infiltration with a resulting fibrosis, hypertrophy and hyperplasia of varying intensity. These processes will gradually change the consistency of the glands from a soft to a fibrous tissue mass. In early and middle age the pressure made on the mandibular lingual plate by these structures may be enough to produce bone resorption (10). The submandibular gland is directly related with the posterior type of SC, the sublingual gland could be related with the anterior type while the paro-tid gland is related with the two types of SC of the mandibular ramus (2).

The surgical removal of the lesion and the posterior histological study are not recommended in the great majority of cases (2,5,8). A panoramic radiography and a CT, can provide sufficient information to make a diagnosis and an adequate follow-up of these patients (8,9). In our opinion, a CT is the most adequate exam to diagnose SC since it is noninvasive and allows to observe the lingual origin of the cavity (2,5,7). In atypical cases or when there is a suspicion of glandular tissue pathology, additional exams may be required (3). A sialography could be useful to visualize the content of the cavity but has several disadvantages like the pain produced by the injection of the contrast agent and the exposure to ionizing radiation (3)

On the other hand, several papers consider the MRI an essential tool in these type of defects, providing multiple imaging planes and different echo sequences (3,11). T1- weighted images are useful for visualizing both salivary gland tissue and to rule out neoplasic changes (12).

To our knowledge, this is the first published case with involvement of both mandibular plates. In fact, in the classification proposed by Arriji et al. (13) this feature is not considered, as the most severe lesions (type III) reach the buccal cortex of the mandible and can cause expansion, but not perforation.

The differential diagnosis in this particular case was more complex and included entities such as the keratocystic odontogenic tumour, giant cells granuloma, hemangioma, arteriovenous malformation, and others malignant pathologies like the ossifying fibrossarcoma or the malignant ameloblastoma, although it must be said that these malignant lesions usually present irregular margins and a fast growth (5,14). Intraosseous salivary gland tumors were also included in the differential diagnosis. However, according to Ojha et al. (15) in these pathologies there should be evidence of osteolysis with integrity of the cortical plates, which was not the case. MRI was particularly useful in the presented patient since it showed that the mandibular bone cavity was filled with tissue that was continuous and identical in signal with that of the submandibular gland.

Most of the studies published to date indicate that SC is asymptomatic and nonprogressive, and therefore a conservative approach based in radiological follow-ups is the most suitable approach. A surgical procedure should only be considered in patients with symptoms or when the findings are not consistent with these defects (2,5,7).
